# Screening for osteogenic activity in extracts from Irish marine organisms: The potential of *Ceramium pallidum*

**DOI:** 10.1371/journal.pone.0207303

**Published:** 2018-11-28

**Authors:** Matthew A. Carson, John Nelson, M. Leonor Cancela, Vincent Laizé, Paulo J. Gavaia, Margaret Rae, Svenja Heesch, Eugene Verzin, Brendan F. Gilmore, Susan A. Clarke

**Affiliations:** 1 School of Nursing and Midwifery, Queen’s University Belfast, Belfast, United Kingdom; 2 School of Biological Sciences, Queen’s University Belfast, Belfast, United Kingdom; 3 Centre of Marine Sciences (CCMAR), University of Algarve, Faro, Portugal; 4 Department of Biomedical Sciences and Medicine, University of Algarve, Faro, Portugal; 5 Algarve Biomedical Center (ABC), University of Algarve, Faro, Portugal; 6 Marine Institute and Seaweed Research Group, Rinville, Oranmore, Co. Galway, Ireland; 7 Irish Seaweed Research Group, Ryan Institute, National University of Ireland Galway, University Road, Galway, Ireland; 8 Orthopaedic department, Royal Victoria Hospital, Belfast, United Kingdom; 9 School of Pharmacy, Queen’s University Belfast, Belfast, United Kingdom; Ohio State University, UNITED STATES

## Abstract

Extracts and compounds derived from marine organisms have reportedly shown some osteogenic potential. As such, these bioactives may aid in the treatment of musculoskeletal conditions such as osteoporosis; helping to address inefficacies with current treatment options. In this study, 72 fractions were tested for their *in vitro* osteogenic activity using a human foetal osteoblast (hFOB) cell line and bone marrow derived mesenchymal stem cells (MSCs), focusing on their cytotoxic, proliferative and differentiation effects. Extracts dissolved in dimethyl sulfoxide and ethanol showed no significant osteogenic potential. However, two extracts derived from powder residues (left over from original organic extractions) caused a significant promotion of MSC differentiation. Bioactivity from powder residues derived from the epiphytic red algae *Ceramium pallidum* is described in detail to highlight its treatment potential. *In vitro*, *C*. *pallidum* was shown to promote MSC differentiation and extracellular matrix mineralisation. *In vivo*, this extract caused a significant increase in opercular bone growth of zebrafish larvae and a significant increase in bone density of regenerated adult caudal fins. Our findings therefore show the importance of continued screening efforts, particularly of novel extract sources, and the presence of bioactive compounds in *C*. *pallidum* extract.

## Introduction

Both disability and mortality associated with osteoporosis are highly significant in developed countries; exceeding the disease burden of common neoplastic disorders in Europe, except for lung cancer [1]. Osteoporosis is associated with decreased bone formation by osteoblasts and increased bone resorption by osteoclasts, which lowers bone mass, reduces mineral content and causes a breakdown of bone microarchitecture [2–4]. This results in weaker bone which is more susceptible to fracture damage, particularly in postmenopausal women. All currently available treatment options for osteoporosis have a variety of issues surrounding their efficacy and suitability for long-term use. For example, bisphosphonates—a commonly prescribed antiresorptive drug—are very effective at reducing fracture risk [5,6]. However, these drugs mainly serve to limit further bone loss and cause only small increases in mineral density (2% or less) [7], whilst also being associated with rare but severe side effects [8]. Alternatively, anabolic treatments are less well developed, with teriparatide the only established treatment option. Unfortunately, the daily injections required with this treatment are very expensive and need to be maintained to aid long term bone formation [8,9]. As such, there is a need for novel osteogenic treatments, which would ideally stimulate the recovery of bone mineral density and structure. In addition to osteoporosis there are numerous other musculoskeletal conditions, such as complex fracture and osteopetrosis, which would also benefit from new effective treatments.

Marine organisms are a particularly interesting resource for novel drugs. These organisms are subjected to an extreme range of environmental variables, which fuels their specialisation and adaptation [10], along with the production of novel molecules/compounds. However, screening efforts for most species remain in their infancy and the search for bioactives remains firmly focussed in the anti-microbial and anti-cancer arenas. Despite this, there are still promising examples of marine invertebrate derived extracts and compounds which show osteogenic activity. One pivotal example is nacre, the lustrous aragonitic layer of molluscan shells, which was shown to significantly aid bone reconstruction of maxillary defects when mixed with the blood of female patients [11,12]. This discovery helped to fuel interest in marine bioactives, though current focus in this area has shifted onto other taxonomic groups. For instance, algae show great bioactive diversity and excellent treatment potential, with fucoidan a particularly well studied example. Derived from multiple taxa, such as brown macroalgae, this highly sulphated and fucose rich polysaccharide inhibits osteoclast bone resorption and promotes osteoblast activity, by activating ERK and JNK pathways [13]. As such, fucoidan shows osteogenic potential both *in vitro* and *in vivo* [14]–though it’s *in vivo* effects remain to be described in detail.

In this study we aimed to address the limitations of current screening efforts by assessing osteogenic activity from extracts of Irish marine organisms. These were derived from a wide range of taxonomic groups–sampled from both shallow and deep-water environments on the west coast of Ireland. Our hypothesis was that osteogenic bioactives were present in one or more of the marine extracts tested in this study, with algal extracts being a likely candidate. This is because previous literature has indicated algal extracts contain bioactive compounds such as fucoidan [14] from brown macroalgae, floridoside [α-D-galactopyranosyl-(1–2)-L-glycerol] from the red algae *Laurencia undulata* and mineralogenic compounds from green macroalgae [15]. However, a diverse range of samples was tested, as research in this field is still in its infancy and it was not known which species/groups would show greatest activity. Extracts were dissolved in either dimethylsulfoxide (DMSO), ethanol or via an alkaline extraction and their proliferative and differentiative potential were assessed *in vitro* using human bone-derived cell systems. One promising extract from the red algae *Ceramium pallidum* (Naegeli ex Kuetzing) Maggs & Hommersand was tested in more detail, including *in vivo* through operculum bone growth and caudal fin regeneration zebrafish models. It is hoped that bioactives from this extract may be a future treatment option for musculoskeletal disorders such as osteoporosis, leading to improved clinical outcomes and a better standard of living.

## Materials and methods

### Sample collection and extraction

Collection: due to the large number of fractions assessed in this study, sampling took place over a prolonged time-period at multiple sites. Specimens were gathered as part of the Beaufort Marine Biodiscovery Research programme, using a previously described strategy [16]. The sample of *C*. *pallidum*, growing epiphytically on the brown alga *Himanthalia elongata* (L.) S.F.Gray, was collected on 05.06.2012 in the lower intertidal at Finavarra, Co. Clare, Ireland. Sample processing and genetic identification followed methods given in Carson *et al*. [17]. A voucher specimen was deposited at the herbarium of the National University of Ireland Galway under the accession number GALW16211. The *rbc*L gene sequence of this specimen is accessible from European Nucleotide Archive ENA or GenBank.

Original extraction: during extraction each specimen was processed using a modified in-house method [16]. Firstly, each specimen was freeze-dried and milled into a fine powder. Five grams of milled material was then mixed with 500 ml of dichloromethane (DCM), at 20°C for 24 h. This suspension was filtered, and any remaining undissolved material was mixed with 500 ml of methanol for a further 24 h at 25°C. This solution was also filtered and the remaining powder residue collected, before the dissolved portions from each extraction were left to air-dry. DCM and methanol portions were then mixed together, whilst the powder residue was kept separately.

**Fig 1:**
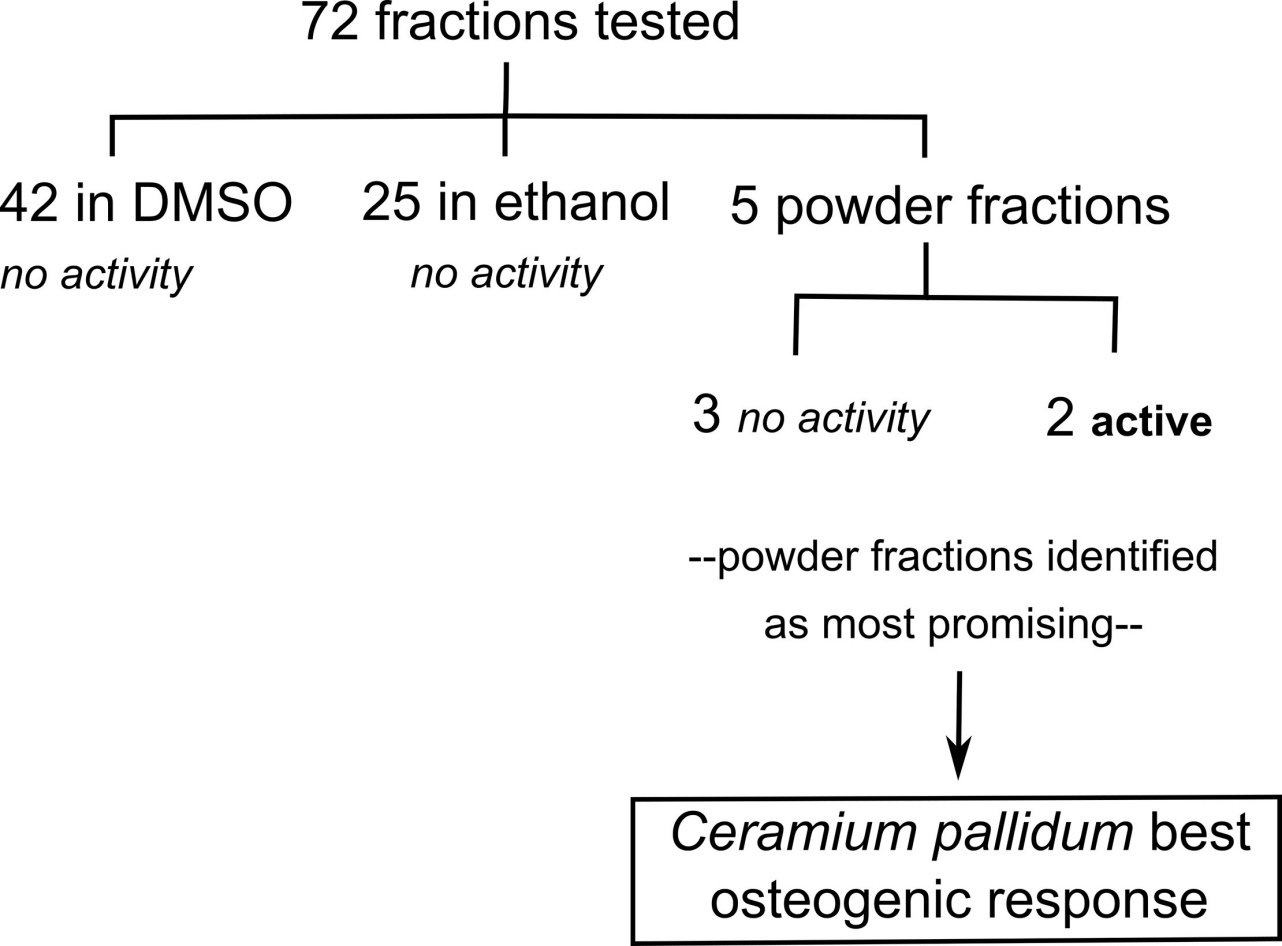
Flow diagram of extracts tested. Diagram showing the results obtained from the screen of 72 extracts. Activity was defined as a significant increase in proliferation or differentiation, which lead to powder extracts being identified as the most promising extract reserve. *Ceramium pallidum* is highlighted, as powder extract from this species gave the best osteogenic result to date (increasing cell differentiation and cell/zebrafish mineralisation); as such it is discussed in greater detail in the present study.

Secondary processing: DCM/methanol mixture was processed further by the addition of either dimethylsulfoxide or ethanol at a concentration of 30 mg/ml. Extracts were then added to culture media at a concentration of 0.1% for DMSO and 0.5% for ethanol. This gave final extract concentrations for all assays ranging between 20 and 31.7 μg/ml for those dissolved in DMSO and 150 μg/ml for those in ethanol (except for E-19: 20.8 μg/ml and E-22: 86 μg/ml). Culture media was filtered (0.22 μm) before use. For the powder residue fractions, extracts were either dissolved in water or a basic extraction was performed, based on a method adapted from previous studies [18,19], via the addition of 0.1 M NaOH (30 mg/ml). Samples then underwent 3 rounds of vortexing, sonication (25 min, 25°C) and mixing (45 min, 37°C). Afterwards, each was centrifuged twice, for 15 min at 3000×g, to separate out supernatant from remaining undissolved powder. Supernatant was neutralised to approximately pH 7.5, via addition of 0.5 M HCl. This extract solution was subsequently added to culture media (or zebrafish system water), which was filtered (0.22 μm) before use. The concentration of powder solutions in culture media or system water ranged between 0.1 and 10%, giving *C*. *pallidum* extract concentrations of 15–1520 μg/ml. See Table 1 for a full list of those extracts tested in this study and Fig 1 for a summary.

**Table 1:** Summary of tested extracts/fractions.

Fraction			Concentr-	Extract effect on cell activity (hFOBs, MSCs)		
number	Common name	Genera/Species/description	ation (μg/ml)	Cytotoxicity	Proliferation	Differentiation
			*DMSO dissolved extracts*			
D-01	Red algae	*Bonnemaisonia hamifera*	5.83	Non-toxic	Small decrease	None
D-02	Coastal sponge	*Porifera demospongiae*	5.97	Non-toxic	None	* *
D-03	Brown algae	*Bifurcaria bifurcata*	5.82	Non-toxic	None	* *
D-04	Coastal sponge	Porifera; Order Poecilosclerida	6.34	Non-toxic	None	* *
D-05(1)	Coastal sea squirt	Chordata; Class Ascidiacea	5.80	Non-toxic	None	* *
D-06	Coastal sponge	*Halichondria panicea*	5.87	Non-toxic	None	* *
D-07	Red algae	*Anotrichium barbatum*	5.71	Non-toxic	None	* *
D-08	Star fish	*Asterias rubens*	6.00	Non-toxic	None	None
D-09(2)	Coastal sea squirt	Chordata; Class Ascidiacea	5.66	Non-toxic	Small decrease	Small decrease
D-10	Coastal sponge	*Haliclona indistincta*	5.76	Non-toxic	None	* *
D-11	Red algae	*Griffithsia corallinoides*	5.84	Non-toxic	None	* *
D-12	Red algae	*Bonnemaisonia hamifera*	6.12	Non-toxic	None	* *
D-13	Coastal sponge	Porifera; Order Poecilosclerida	6.16	Non-toxic	None	* *
D-14	Coastal sponge	Dictyoceratid sponge	5.80	Non-toxic	None	* *
D-15	Green algae	*Ulva linza*	5.76	Decreased	None	Small increase
D-16	Brown algae	*Cystoseira tamariscifolia*	5.80	Decreased	None	* *
D-17	Red algae	*Polysiphonia elongata*	6.12	Decreased	Small decrease	None
D-18	Brown algae	*Sargassum muticum*	6.11	Decreased	None	* *
D-19	Coastal sponge	Porifera; Family Suberitidae	5.96	Decreased	None	* *
D-20	Brown algae	*Desmarestia aculeata*	5.90	Decreased	Small decrease	Increased
D-21	Red algae	*Polysiphonia stricta*	5.72	Decreased	Small decrease	Decreased
D-22	Coastal sponge	*Polymastia boletiformis*	5.98	Decreased	None	* *
D-23	Brown algae	*Sargassum muticum*	5.85	Decreased	None	* *
D-24	Coastal sponge	*Suberites ficus*	6.03	Decreased	Small decrease	Decreased
D-25	Coastal sponge	*Dysidea fragilis*	5.91	Decreased	None	None
D-26(1)	Tunicate	Ascidian; Family Styelidae	6.03	Decreased	None	Increased
D-27	Coastal sponge	*Haliclona simulans*	6.04	Decreased	Small decrease	None
D-28(2)	Tunicate	Ascidian; Family Styelidae	5.84	Decreased	Small decrease	None
D-29	Coastal sponge	*Haliclona indistincta*	6.23	Decreased	Small increase	* *
D-30	Brown algae	*Cystoseira baccata*	5.70	Decreased	Small increase	* *
D-31	Brown algae	*Desmarestia viridis*	6.06	Decreased	None	* *
D-32	Brown algae	*Desmarestia ligulata*	6.25	Decreased	Small increase	None
D-33	Coral	Soft coral	5.36	Decreased	Small increase	None
D-34	Hydrozoan	Hydrozoan	4.00	Decreased	None	None
D-35	Coastal sponge	Sea fan; Paramuricea	6.06	Decreased	None	* *
D-36	Sea star	Sea star	6.07	Decreased	Small increase	None
D-37	Brittle star	N/A	5.33	Decreased	Small increase	None
D-38	Brown algae	*Cystoseira foeniculacea*	6.20	Decreased	Small increase	* *
D-39	Brown algae	*Cystoseira nodicaulis*	6.04	Decreased	None	None
D-40	Deep sea sponge	*Lissodendoryx diversichela*	5.67	Decreased	None	None
D-41	Sea cucumber	N/A	6.00	Decreased	None	
D-42	Sea fan	N/A	6.00	Decreased	None	
			*Ethanol dissolved extracts*			
E-01	Star fish	N/A	150	Small increase	Decreased	Decreased
E-02	Sea fan	N/A	150	Non-toxic	None	
E-03	Deep sea sponge	*Haliclona simulans*	150	Non-toxic	None	
E-04	Soft coral	Soft coral	150	Increase	Decrease	Decreased
E-05	Sea star	Sea star	150	Non-toxic	None	Small increase
E-06	Sea star	Cushion star fish	150	Non-toxic	Decreased	
E-07	Sea star	N/A	150	Non-toxic	None	
E-08	Sea star	Sea star	150	Non-toxic	Decreased	
E-09	Sea pen	Sea pen	150	Non-toxic	None	
E-10	Sea star	Cushion sea star	150	Non-toxic	None	
E-11	Sea fan	*Phanophathes sp*.	150	Non-toxic	None	Decreased
E-12	Red algae	*Corallina elongata*	150	Non-toxic	None	
E-13	Algae (griffithsia)	*Griffithsia corallinoides*	150	Non-toxic	None	
E-14	Sea star	Cushion sea star	150	increase	None	
E-15	Sea fan	*Pentaculacea*	150	Non-toxic	None	
E-16	Deep sea sponge	*Haliclona indistincta*	150	Non-toxic	Decreased	
E-17	Red algae	*Ceramium secundatum*	150	Non-toxic	Decreased	Decreased
E-18	Brown algae	*Bifurcaria bifurcata*	150	Toxic	Decreased	
E-19	Red algae	*Boergeseniella fructiculosa*	20.8	Non-toxic	Decreased	None
E-20	Red algae	*Plocamium cartilagineum*	150	Increase	Decreased	Decreased
E-21	Red algae	*Osmundea sp*.	150	Decreased	None	Decreased
E-22	Brown algae	Ectocarpales (Puncatria)	86	Decreased	None	Decreased
E-23	Sponge	*Cladostephus spongiosus*	150	Non-toxic	Decreased	
E-24	Coral	N/A	150	Non-toxic	Decreased	Decreased
E-25	Deep sea sponge	*Haliclona indistincta*	150	Decreased	None	Decreased
			*Powder residue extracts*			
P-01	Coral	Lophelia coral			1 small decrease	None
P-02	Seaweed	*Percursaria percursa*			1 small decrease	Increased
P-03	Red algae	*Porphyra umbilicalis*			1 small decrease	Small decrease
P-04	Deep sea sponge	*Psolus squamatus*			1 small decrease	Increased
P-05	Red algae	*Osmundea sp*.			1 small decrease	Decreased
P-06	Red algae	*Ceramium pallidum*			Variable	Increased

‘D’ indicates a fraction/extract dissolved in DMSO, ‘E’ indicates dissolved in ethanol and ‘P’ indicates a powder residue. (1) and (2) indicate separate samples from the same species.

### Cell culture–hFOB and MSC

**Fig 2:**
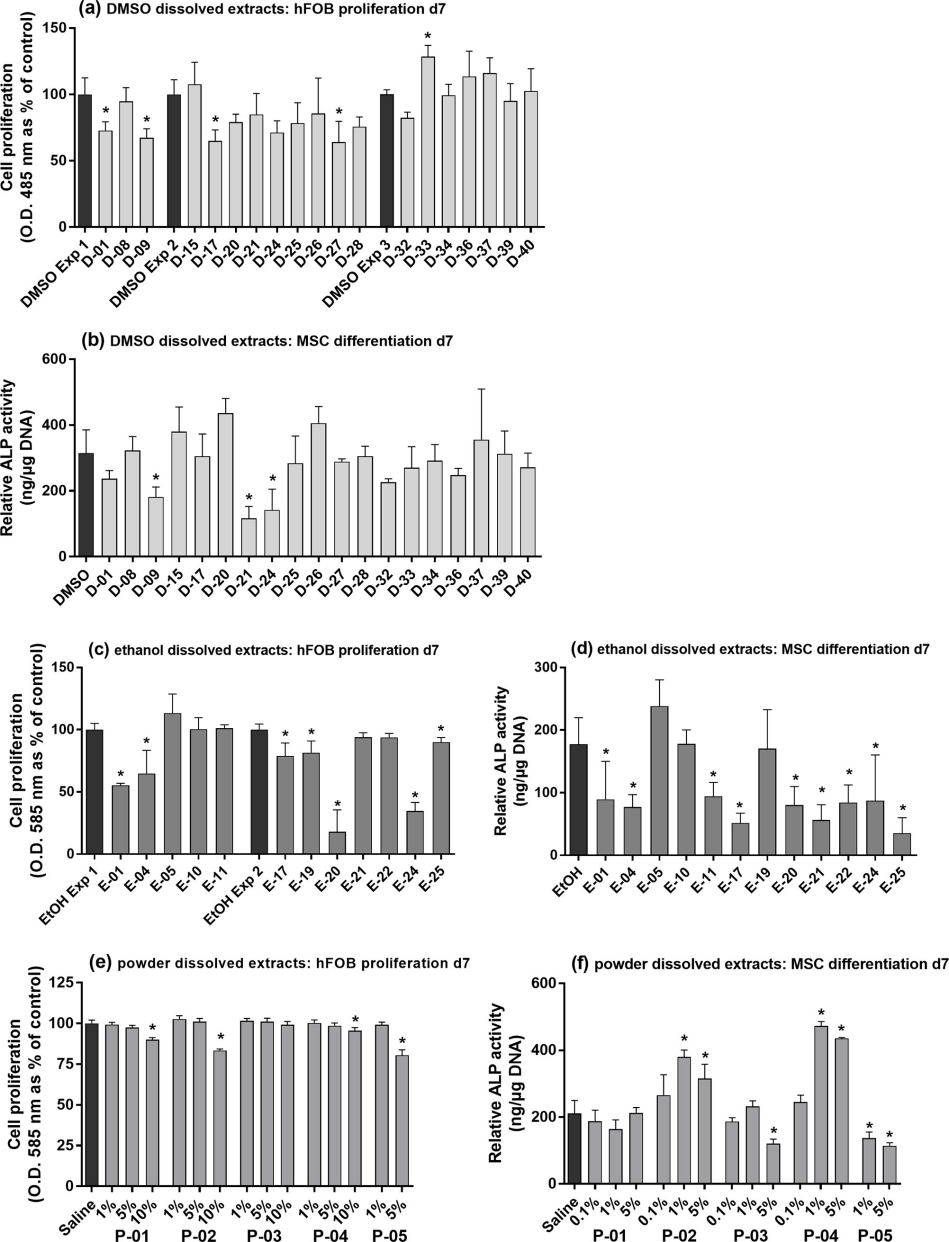
*In vitro* effects of various extracts. **(a, c, e)** Proliferation of hFOBs at day 7 determined by PicoGreen assay. **(a)** Cells were challenged with either DMSO dissolved fractions D-01 to D-42 (subset shown in figure, complete set presented in S1, S2 and S3 Figs) or control (0.1% DMSO). **(c)** Cells were challenged with ethanol dissolved extracts E-01 to E-25 (subset shown in figure, complete set shown in S5 and S6 Figs) or control (0.5% EtOH). **(e)** Cells were challenged with control (saline solution–average of proliferation values for treatments between 1–10% concentration) or powder extracts P-01 to P-05, dissolved in a saline solution and at concentrations in cell media of 1, 5 or 10%. Cell proliferation, as a percentage of control, is presented as mean +/- standard deviation (SD), (n = 3 or 4). **(b, d, f)** ALP activity of MSCs at day 7 normalised to DNA concentration, treated with the same extract subset or control as detailed previously for DMSO **(b)**, ethanol **(d)** and powder extracts **(f)**. Relative ALP activity is presented as the mean +/- SD (n = 3). * indicates a statistically significant difference (*p*<0.05) compared to the relevant control–**(a)** included 3 separate controls as it combines data from 3 individual experiments.

**Fig 3:**
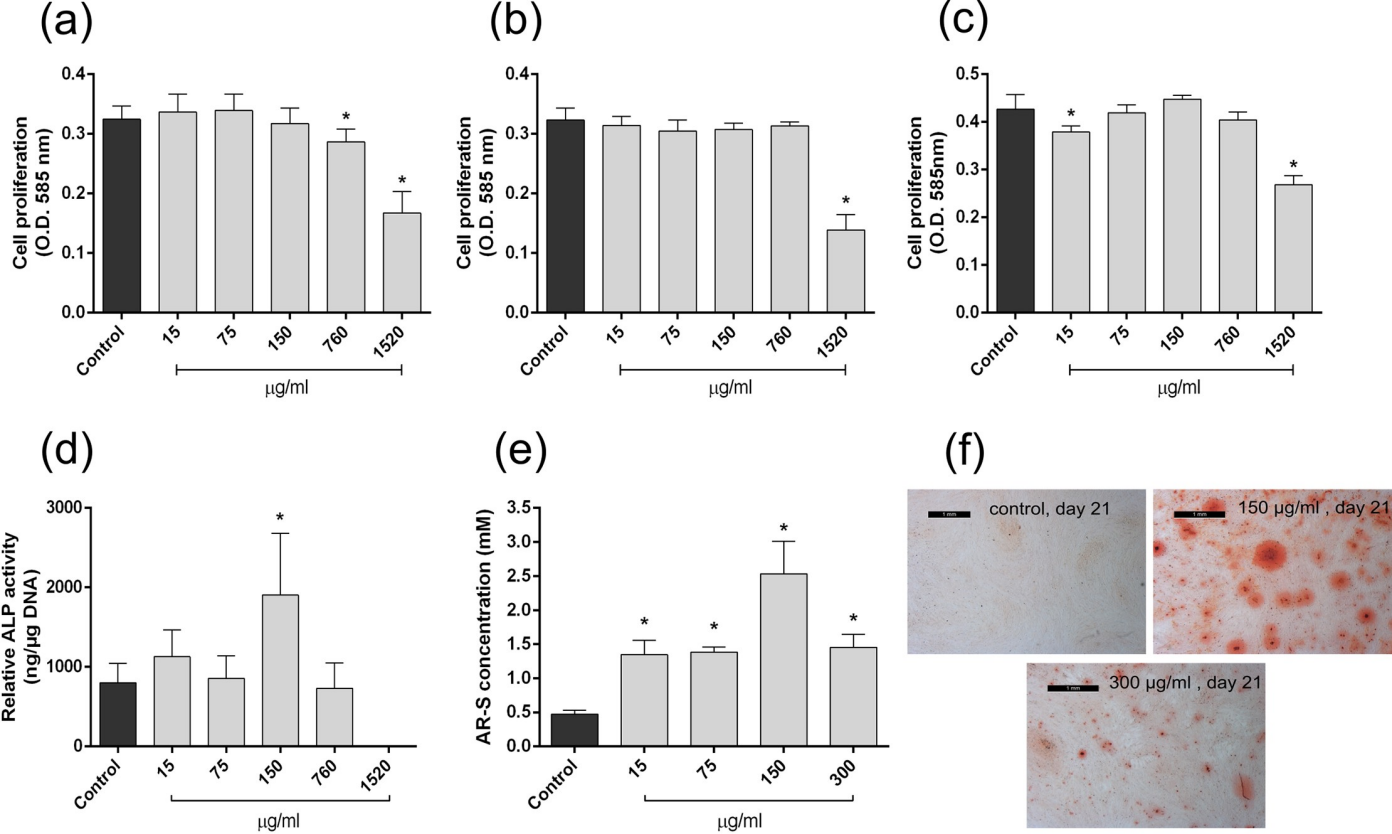
*In vitro* osteogenic effects of *C*. *pallidum* extract. **(a)**-**(c)** Proliferation of MSCs at day 1 **(a)**, 4 **(b)** and 7 **(c)** using crystal violet assay. Cell number is presented as mean +/- SD, (n = 4). **(d)** Differentiation of MSCs at day 14, presented as relative ALP activity, normalised to DNA content–mean +/- SD (n = 4). **(e)** *In vitro* mineralisation of MSCs at day 21. AR-S concentration is presented as mean +/- SD (n = 4). **(f)** Micrographs of MSC monolayers stained with AR-S, for control day 21 and *C*. *pallidum* at a 150 and 300 μg/ml concentration (black rectangle is a 1 mm scale for each micrograph). * indicates a statistically significant difference (*p*<0.05) compared to the relevant control.

The hFOB 1.19 (ATCC CRL-11372) cell line was cultured in T75 flasks and expanded by subculturing (0.25% Trypsin EDTA, 1:4 slitting ratio). hFOBs were cultured in complete media comprised of DMEM/HAM F12 (Sigma-Aldrich, UK) supplemented with 10% foetal bovine serum (Sigma-Aldrich, UK) and 2 mM L-glutamine (PAA, UK). Cells were used between passages 6 and 10. MSCs were extracted from vertebral bone marrow samples collected by surgeons at the Royal Victoria Hospital, Belfast, during the placement of intervertebral screws. After collection, samples were processed to extract the white blood cell-containing component, which was subsequently cultured until 75% confluent. MSCs were cultured in α-MEM (Thermo Fisher Scientific, UK) supplemented with 10% foetal bovine serum, 2 mM L-glutamine and 100 U/ml pen-strep (all sourced from Thermo Fisher Scientific, UK). After initial growth, MSCs were expanded by subculturing (using 0.25% Trypsin-EDTA and a 1:4 splitting ratio); no cells beyond passage 6 were used in experiments (see Figs 2 and 3 for results using these cells).

### Cytotoxicity–lactate dehydrogenase (LDH)

The CytoTox 96 Non-Radioactive Cytotoxicity Assay kit (Promega, UK) was used to quantify levels of lactate dehydrogenase (LDH), an enzyme normally constrained to the cytoplasm of healthy cells. Its release in the culture medium gives an indication of extract cytotoxicity. hFOBs were plated at a density of 1 x 10^4^ cells/cm^2^ and were given a 24 h attachment period and 24 h with extract treatments, before conditioned medium was removed and LDH level quantified according to the manufacturer’s protocol.

### Cell proliferation–crystal violet

Extract concentrations were as follows: 1. 150 μg/ml (E-19: 20.8 μg/ml and E-22: 86 μg/ml) for ethanol dissolved extracts and 2. 15, 75, 150, 760 and 1520 μg/ml for *C*. *pallidum* powder extract. Controls were as follows: 1. 0.5% ethanol and 2. 1, 5 or 10% saline solution (control presented as an average of these three treatments to ensure saline addition had no significant effect). hFOBs were plated at a density of 1 × 10^4^ cells/cm^2^ and MSCs at 2 × 10^4^ cells/cm^2^. Subsequently, each cell type was challenged by the extracts for 1, 4 or 7 days. Seven-day cultures were re-fed once with treated media on day 4. Crystal violet staining was used to measure cell proliferation, in a method similar to that of Kim et al. [13]. Briefly, cells were fixed at 25°C for 30 min by the addition of 100 μl of 2% paraformaldehyde (Sigma-Aldrich, UK) solution (pH 6.8 in PBS). Cell monolayers were stained at 25°C for 30 min, using 100 μl of crystal violet solution (Sigma-Aldrich, UK) per well (0.1% in dH_2_O, filtered before use). Dye was extracted from monolayers by the addition of 100 μl of 1 M acidified methanol. Treatments were blanked using acidified methanol and absorbance was measured at 585 nm, using a Multiskan Spectrum microplate reader (Thermo Fisher Scientific, UK).

#### Cell proliferation–PicoGreen

The Quant-iT PicoGreen dsDNA Assay kit (Thermo Fisher Scientific, UK) was used to quantify the amount of double stranded (ds) DNA present within a sample, following manufacturer’s instructions, to determine proliferation for DMSO dissolved extracts and to normalize alkaline phosphatase (ALP) results. For proliferation of DMSO extracts concentrations ranged between 20 and 31.7 μg/ml, whilst control was 0.1% DMSO, with a separate control included on each assay plate used (large extract number). Cells were challenged by extracts for 1, 4 or 7 days, medium was removed and the cells were washed twice with PBS or alkaline buffer solution (5 M NaCl, 1 M Tris-Cl pH 9.5, 1 M MgCl_2_). 250μl of lysis buffer (0.1 or 0.2% Triton X-100 (Sigma-Aldrich, UK) in PBS or alkaline buffer) was added to each well which was then subjected to at least 1 cycle of freeze (-80°C) and thaw (+37°C). This ensured that cell membranes were completely lysed through ice crystal formation, therefore releasing all dsDNA into solution. Sample wells were then mixed and 50 μl of each lysate was added to assay plates in duplicate. A standard curve of 1 mg DNA/ml, 100 ng/ml, 10 ng/ml, 1 ng/ml and 0 ng/ml was added in duplicate to each assay plate. Finally, 50 μl of test solution was added to all wells and fluorescence levels were read at excitation 480 nm, emission 520 nm using a GENios microplate reader (TECAN, Austria).

#### Cell differentiation

MSCs were plated at a density of 1 × 10^4^ cells/cm^2^ in 96-well plates and cultured with extracts dissolved in osteogenic media (complete media supplemented with 50 μM ascorbate-2-phosphate, 10 μM β-glycerol phosphate and 0.01 μM dexamethasone, all sourced from Sigma-Aldrich, UK) for 7 or 14 days. Extract concentrations and control solutions were the same as those detailed previously, except for the use of osteogenic (rather than complete) media. Extract containing media was replaced twice weekly. Alkaline phosphatase (ALP) activity was measured following *p*-nitrophenyl phosphate level. Briefly, at each time point cells were: 1. washed with an alkaline buffer solution (5 M NaCl, 1 M Tris-Cl pH 9.5, 1 M MgCl_2_), 2. lysed by addition of 250 μl of alkaline buffer containing 0.2% Triton X-100, 3. left to gently mix for 20 min on ice and 4. stored at -80°C. Upon defrosting, 50 μl from each cell extract was added to a test plate in duplicate and supplemented with 200 μl of conditioned medium, consisting of alkaline buffer solution (Sigma-Aldrich, UK) and *p*-nitrophenyl phosphate substrate (Sigma-Aldrich, UK). Each test plate was then covered in foil and incubated at 37°C for 30 min, allowing the coupled enzymatic reaction to proceed. The reaction was stopped by the addition of 50 μl of 3 M NaOH to each well, before absorbance was read at 450 nm using a GENios microplate reader (TECAN, Austria). Finally, ALP activity was normalised to DNA concentration, determined via PicoGreen assay to account for variations in cell proliferation.

#### Extracellular matrix mineralisation

MSCs were grown to 75% confluency in 24-well plates, before osteogenic media and *C*. *pallidum* extracts at 15, 75, 150 and 300 μg/ml concentrations were added—for 21 days. Medium was replaced twice weekly. Alizarin red S (AR-S; Sigma-Aldrich, UK) staining was used to detect mineralisation of the extracellular matrix produced by mature osteoblasts. At each time point, cells were washed 3x with PBS and fixed with 4% paraformaldehyde (0.25 ml) at 25°C for 1 h. Fixative was then removed and wells washed 3x with dH_2_O. 40 mM AR-S, adjusted to pH 4.2 using ammonium hydroxide, was added to each well (0.5 ml). Plates were left to stain at 25°C for 15 min, with gentle agitation on an orbital shaker. Staining solution was discarded and each well was washed again, 4x with dH_2_O, before being left to air-dry. Finally, wells were de-stained using a solution of 10% cetylpyridinium chloride (Sigma-Aldrich, UK) in sodium phosphate (pH 7.0). A 100 μl aliquot of each treatment was collected, transferred to a 96-well plate and then used for measuring absorbance at 550 nm. Absorbance readings were then converted to AR-S content values via a standard curve equation (R^2^ = 0.99) made using a dilution series of AR-S stock solution.

#### *in vivo* operculum model

**Fig 4:**
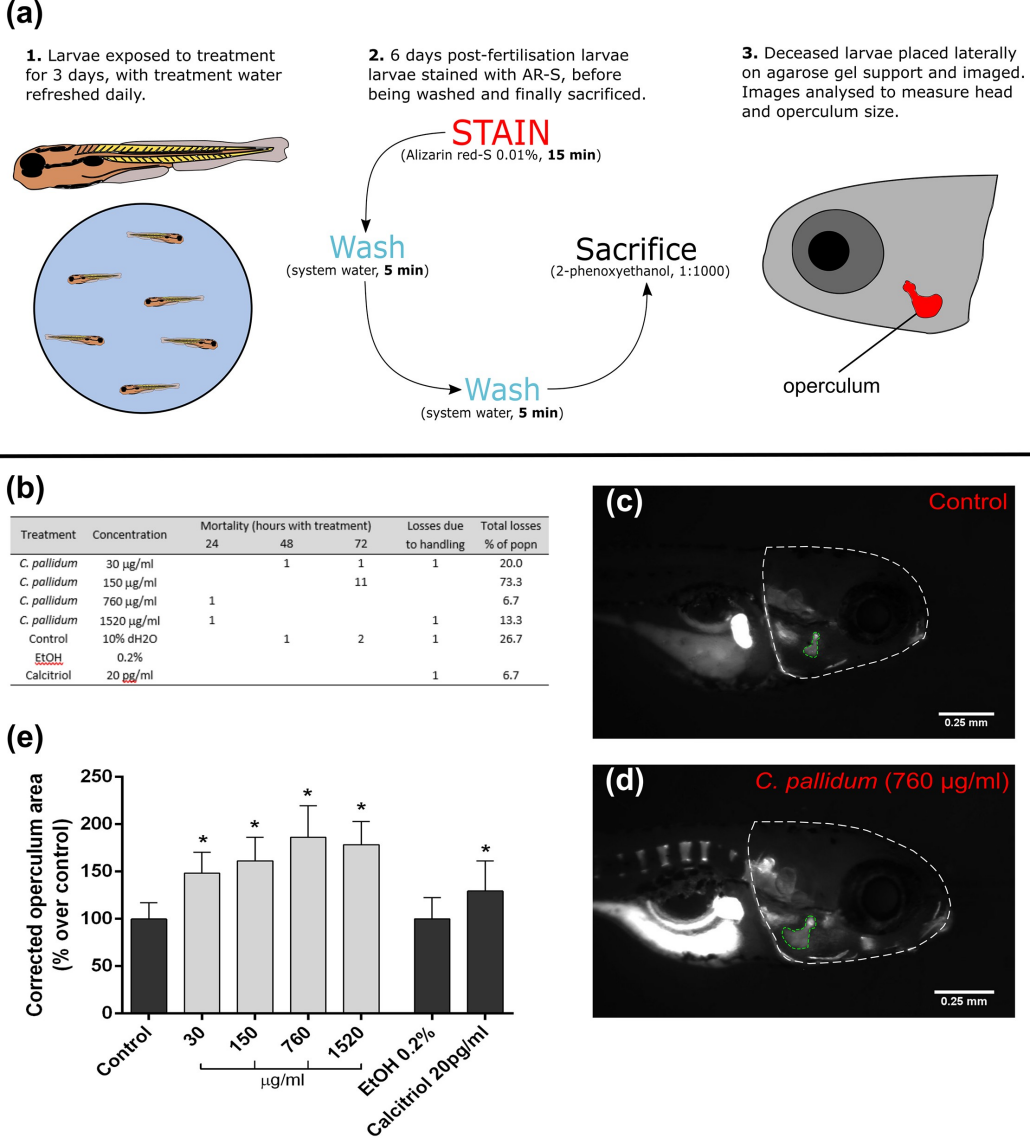
*In vivo* osteogenic effects of *C*. *pallidum* extract–operculum model. **(a)** Schematic detailing the method used for the operculum bone growth model. **(b)** Mortality rates of zebrafish larvae during *in vivo* experimentation. **(c)** Picture of a control alizarin red-S stained zebrafish larvae at 6 dpf showing head and operculum area evaluated through morphometric analysis. **(d)** Picture of a stained larvae treated with 760 μg/ml *C*. *pallidum*. **(e)** Operculum growth for *C*. *pallidum* treatments. Control was fish water with 10% dH2O. A 0.2% EtOH control corresponded to the positive calcitriol control. Corrected operculum area is presented as mean +/- SD (n = 15).

For a more detailed method see Tarasco *et al*. [20] (see Fig 4 for visualisation of method). Briefly, sexually mature AB wild-type strain zebrafish were mated and fertilised eggs were collected and transferred to a 1-L container. Eggs were then placed in an incubator (28.0 ± 0.1°C) for 72 h with a photoperiod of 14–10 h light-dark. At three days post-fertilisation (dpf), viable larvae were transferred into 6-well plates. Each well housed 15 larvae in 10 ml of system water, to which extracts were added. Concentrations of *C*. *pallidum* extract included 30, 150, 760 and 1520 μg/ml. Controls included a standard control group (plain water), a positive control of 20 pg/ml calcitriol (Tocris Bioscience) dissolved in ethanol, as well as a negative control for this group—containing only 0.2% ethanol. 70% of treatment water was replaced daily during the three-day exposure period. Subsequently, larvae were stained in excess AR-S solution (0.01%, pH 7.4) for 15 min, washed twice in system water (5 min) and then sacrificed (1:1000 dose of 2-phenoxyethanol; Sigma-Aldrich, UK). Larvae were sacrificed in batches of 15 individuals and imaged directly after euthanasia (via lateral placement on a 2% agarose gel). An MZ 7.5 stereomicroscope (Leica, Wetzlar, Germany) was used, equipped with a green light filter (λ_ex_ = 530–560 nm and λ_em_ = 580 nm) and a black-and-white F-View II camera (Olympus, Hamburg, Germany). Images were subsequently analysed using Image J 1.49 (Wayne Rasband, Bethesda) software. Ratios comparing the head and operculum area were then calculated and given as a percentage of the control.

#### *in vivo* caudal fin regeneration model

**Fig 5:**
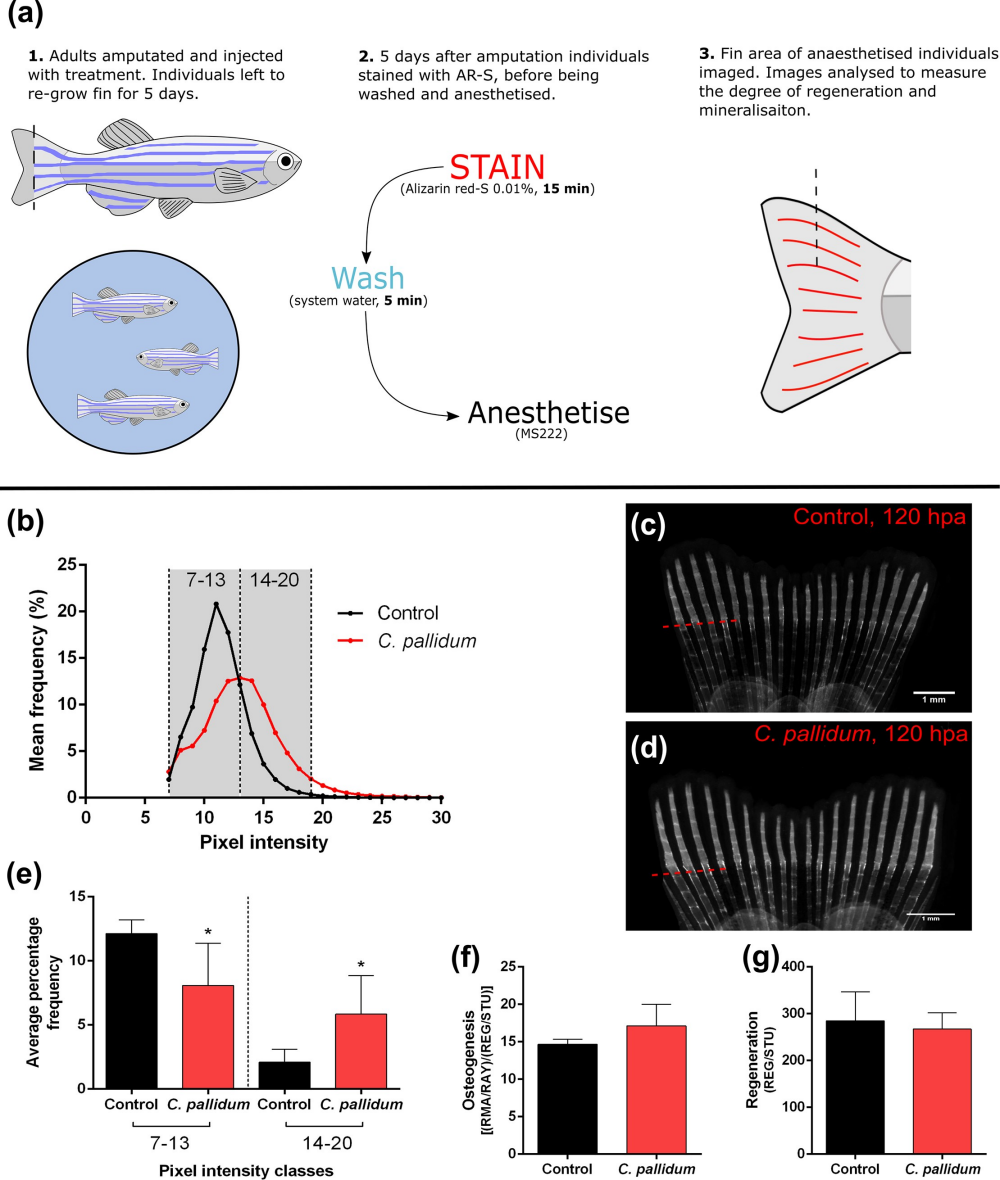
*In vivo* osteogenic effects of *C*. *pallidum* extract–caudal fin model. **(a)** Schematic detailing the method used for the caudal fin model. **(b)** Histogram showing pixel intensity frequencies in classes 7–13 and 14–20. **(c)** Picture of alizarin red-S stained caudal fin (red channel) from a control individual 120 hpa. **(d)** Picture of stained caudal fin from an individual treated with *C*. *pallidum* at 1.56 μg per mg fish weight (same for all treated individuals), 120 hpa. **(e)** Average percentage frequency (n = 5) for control and *C*. *pallidum* treated individuals within pixel intensity classes of 7–13 and 14–20. **(f)** Degree of osteogenesis at 120 hpa. **(g)** Degree of regeneration at 120 hpa. * indicates a statistically significant difference (*p*<0.05) compared to the relevant control.

Caudal fin regeneration experiments used a method modified from Cardeira *et al*. [21] (see Fig 5 for visualisation of method). Adult wild-type AB zebrafish aged 3–5 months were used in all experiments. Individuals were anesthetised via submersion in a water bath containing MS222 (Sigma-Aldrich, St. Louis, MO) and subsequently quickly dried and weighed. Injection volumes were calculated using each individual weight, based on a target addition of 3 μl solution per 100 mg fish weight. Treatment solutions included either a saline solution (control) or a saline dissolved mixture of *C*. *pallidum* powder residue at a concentration of 1.56 μg per mg fish weight. Individuals were then amputated 1–2 segments anterior to the bifurcation of the most peripheral branching lepidotrichia. After amputation, treatments were administered via intraperitoneal injection and individuals were then placed into plastic containers holding 900 ml of water, at a density of five fish per container. Container water was replaced daily and heated to 33°C to accelerate the regenerative process [22] and fish were fed twice daily with dried food. After a five-day period, individuals were live-stained by submersion in 200 ml of 0.01% AR-S solution for 15 min, then washed for 5 min before being anesthetized in a solution of MS222 [23]. Individuals were then immediately imaged, using the same camera system as described for the larvae. Fluorescence and bright field images were collected for each individual, using an exposure of 500 ms. Images were then analysed using Image J 1.49 software, allowing a number of measurements to be taken. Total regenerated area (REG) was measured from the amputation plane to distal end of the fin and was corrected with the stump width (STU). Mean ray width (RAY) was determined as the mean width of rays at the first intersegment joint below the amputation plane. Real mineral area (RMA) was measured after applying a colour threshold which selected only the mineralised red areas. Finally, the intensity of calcium staining was assessed using the intensity of pixels within mineralised regions (using the YUV colour model and intensity values in the 15–254 arbitrary unit range).

#### Ethical approval

All methods involving human tissue samples were carried out in accordance with the Human Tissue Act. Ethical approval was granted by the North East-Tyne and Wear South Research Ethics Committee (REC ref 15/NE/0250), specifically for the use of human derived tissue from NHS patients. Informed consent was obtained from all patients who provided samples for use in this work.

All experiments involving zebrafish were completed in Portugal. Only qualified operators were involved in animal handling and experimentation, having been legally accredited by the Portuguese Direção Geral de Alimentação e Veterinária (DGAV). All experimental procedures involving zebrafish followed the EU (Directive 86/609/CEE and 2010/63/EU) and National (Portaria n.° 1005/92 de 23 de Outubro; Portaria n.° 466/95 de 17 de Maio; Portaria n.° 1131/97 de 7 de Novembro) legislation for animal experimentation and welfare. Experimental procedures involving zebrafish were performed under authorization (0421/000/000) from the DGAV, complying with the decreto de lei 113/2013 de 7 de Agosto, from the Portuguese legislation.

#### Statistical analysis

Prism version 6.00 (GraphPad Software, Inc. La Jolla, CA) software was used for statistical analysis. Datasets were tested for normality using D’Agostino-Pearson and Shapiro-Wilk test. Statistical comparisons were analysed with one-way ANOVA and post-hoc with Dunnett’s multiple comparison test. For zebrafish results the same analysis was conducted, though positive control comparisons were made using unpaired t-tests with Welch’s correction. For all tests, a *p*<0.05 was considered statistically significant. Sample sizes can be found in each figure legend.

## Results

Screening detailed within this manuscript first focused on marine organism extract material dissolved in DMSO, before being expanded to include ethanol dissolved extracts and powder residues. Throughout this process (summarised in Fig 1, more detailed summary in Table 1) powder residue material showed most promise through its effect on cell differentiation. However, *Ceramium pallidum* extract was selected as having the best overall osteogenic potential–increasing cell differentiation and mineralisation, as well as promoting mineralisation *in vivo*. For all assays, time points appropriate to the mechanism being tested were chosen, such as cytotoxicity at day 1 and differentiation at day 7.

### Solvent fractions and powder derived extracts

DMSO was chosen as a solvent for this screen as it is the most commonly used solvent in screening work and is effective at dissolving a wide range of compounds [24]. However, it was limited to a 0.1% concentration to avoid changes in cell membrane properties and cell morphology [25], giving an effective concentration ranging between 20 and 31.7 μg/ml for each extract. Fractions dissolved in DMSO had little effect on hFOB proliferation [Fig 2(A)]. At day 7, fractions D-01, 09, 17 and 27 caused small but significant decreases in cell proliferation relative to control (DMSO), whilst D-33 caused a small significant increase (cell proliferation: 0.71, control: 0.55, *p* = 0.047). This subset reflects the trends of all DMSO dissolved extracts (total of 42), as only small deviations in proliferation were seen for all extracts tested (see S1, S2 and S3 Figs). A similar trend was also seen for day 7 MSC differentiation [Fig 2(B)]; MSCs were used as they were shown during preliminary tests to have a more sensitive differentiation response. There were no significant increases in alkaline phosphatase (ALP) activity, though D-15, 20 and 26 were slightly higher than control. Decreased activity was more common, of which D-09 and particularly D-21 and 24 were significant. Lactate dehydrogenase (LDH) cytotoxicity results (presented in S4 Fig) showed that none of the extracts were toxic, whilst D-15-42 actually caused small decreases in cell death levels.

Ethanol was subsequently chosen for use as it was shown to have less of a toxic effect on hFOBs than DMSO during preliminary testing, being tolerated up to a 0.5% level. This allowed a concentration of 150 μg/ml to be reached for each extract (except for E-19: 20.8 μg/ml and E-22: 86 μg/ml, as less extract material was available for testing). Ethanol dissolved extracts had similar effects as those in DMSO, mainly serving to decrease hFOB cell proliferation [Fig 2(C)]. Of the subset shown, all decreases were significant—including E-01, 04, 17, 19, 20, 24 and 25. Remaining extracts were comparable in value to the control (EtOH), except for E-05 which was slightly increased. This subset again reflects the trends of the whole extract set tested (total of 25; complete set is presented in S5 and S6 Figs). MSC ALP activity [Fig 2(D)] was similar to that of hFOB proliferation, with E-05 again the only extract showing an increase compared to control. E-10 and E-19 had no effect, whilst all remaining extracts caused significant reductions. For cytotoxicity, E-01, 04, 14 and 20 caused small increases in LDH enzyme activity levels, indicating increased cell death, but these were small and therefore unlikely to be toxic (S7 Fig). Alternatively, E-21, 22 and 25 decreased cell death, whilst the remaining treatments were all non-toxic.

Due to the sensitivity of the LDH assay to dissolved particulate matter, cytotoxicity results were not reliable for powder extract treatments and have therefore not been included in the supplementary material. Furthermore, powder extracts had a very limited effect on hFOB proliferation [Fig 2(E)]. All 1 and 5% treatment concentrations were comparable to control, except for P-05 which was significantly reduced at 5% (*p* < 0.0001). Apart from P-03, all other powder treatments significantly reduced proliferation at their maximum concentration of 10%. MSC ALP results used a lower concentration range of 0.1, 1 and 5% [Fig 2(F)]. P-01 had no effect other than a slight reduction in ALP activity, as did P-03 which was significant at 5%; whilst P-05 was significantly reduced at both 1 and 5%. However, extracts P-02 and 04 caused a marked increase in ALP activity at 1 and 5% concentrations. Each peaked at 1%, though P-04 caused a greater effect than P-02—with an approximate 2.5-fold increase in ALP activity compared to control.

### Mineralogenic effects of *C*. *pallidum* powder fraction *in vitro*

Effect of the powder fraction from *Ceramium pallidum* (P-06) on MSC cell proliferation changed slightly between timepoints [Fig 3(A)–3(C)]. At all timepoints (day 1, 4 and 7) the greatest concentration, 1520 μg/ml, caused a significant reduction in proliferation to a level approximately half that of the control. At day 1, cell proliferation was also reduced upon exposure to 760 μg/ml of P-06, though this effect was lost at day 4. By day 7, a bell-shaped response was seen, with the 150 μg/ml concentration stimulating the most cell proliferation, which slightly exceeded that of the control. 15 and 1520 μg/ml concentrations were significantly reduced, though this was most pronounced for the 1520 μg/ml treatment. Also at day 7, ALP activity of MSCs was significantly increased at a 150 μg/ml concentration [Fig 3(D)], whilst remaining concentrations were similar in value to control. 1520 μg/ml was an exception to this, as no ALP activity was detected. Levels of mineralisation, expressed as the amount of alizarin red-S bound to the mineral deposited in the extracellular matrix (ECM) of MSCs, were determined at day 21 [Fig 3(E)]. A clear trend was apparent, as each concentration of *C*. *pallidum* caused a marked significant increase (*p* < 0.0001 for all) in mineralisation level. For the 15, 75 and 300 μg/ml concentrations these increases were almost three times greater than the control value. However, the 150 μg/ml treatment performed best, causing a 5-fold increase in ECM mineralisation.

### Osteogenic effect of *C*. *pallidum* powder fraction *in vivo*

During the treatment period there were small losses of 1 or 2 larvae in each treatment [Fig 4(B)]. For example, 1 sample was lost in each of the 30 and 1520 μg/ml treatment groups, due to damage during placement for imaging. There was also one large mortality event 72 hours after initial exposure in the 150 μg/ml treatment group, whereby 11 larvae died—accounting for 73% of the population (15 larvae total); see discussion for explanation of this.

Overall, *C*. *pallidum* caused a significant increase of larvae operculum growth compared to the control, ranging from 48.4% ± 21.9% at a 30 μg/ml concentration, to a maximum of 86.1% ± 30.0% at a 760 μg/ml concentration [Fig 4(E)]. The highest treatment concentration, 1520 μg/ml, had a slightly lower—but still significant—average size increase of 78.5 ± 23.7%. Calcitriol, as a positive control, significantly increased operculum size in the order of 29.6 ± 31.6%, relative to the 0.2% ethanol vehicle control. Images of stained zebrafish larvae illustrate this effect, highlighting the increase in operculum size with *C*. *pallidum* treatment (760 **μg**/ml) compared to control.

In adults, *C*. *pallidum* extract was used at 1.56 μg per mg fish weight. Extract treatment caused a slight increase in osteogenesis of the regenerating fin area 120 h post-amputation (hpa), though this was not significantly different from control [Fig 5(F)]. Alternatively, regeneration was slightly decreased upon *C*. *pallidum* treatment, though also not to a significant degree [Fig 5(G)]. Pixel intensity formed two very distinct frequency curves between control and *C*. *pallidum* treatments [Fig 5(B)]. For the control, mean frequency peaked in the low intensity pixel classes (7–13), whereas *C*. *pallidum* was spread more evenly–having a greater representation in the high intensity classes (13–19). Comparing the average percentage frequency between the two classes, *C*. *pallidum* was shown to have fewer low intensity pixels and more high intensity ones compared to control, both of which were significantly different [Fig 5(E)].

## Discussion

This study aimed to screen a wide range of marine organisms, sourced from the west coast of Ireland, for their osteogenic activities (as summarised in Fig 1 and Table 1); culminating in the identification of an extract of *C*. *pallidum*, which had strong *in vitro* and *in vivo* mineralogenic effects. The approach used was fairly novel, as screening efforts are still in their infancy and tend to focus on identifying active compounds from one organism [26] or high-throughput screening of many small molecules [27,28]. Specifically, a hFOB cell line was chosen to screen for initial cytocompatibility by determining toxicity and extract effects on cell proliferation. hFOBs benefit from being easily sourced whilst maintaining key osteoblast markers and characteristics [29,30]. However, preliminary testing showed MSCs to have a greater and more sensitive differentiation response than hFOBs, and these were therefore used to determine osteogenic differentiation potential of promising extracts. During the screening process criteria used to identify ‘active’ extracts were lack of toxicity and promotion of osteoblast activity. This promotion could be of either proliferation or differentiation, as either characteristic would be beneficial in osteoporosis or fracture treatment; by increasing the size of the active cell component or promoting cell maturation. Extracts were also deemed active if decreased proliferation was coupled with increased differentiation. This is because osteoblasts undergo a sequential development, reducing their proliferation during differentiation towards the mature phenotype [31].

The first 42 extracts screened covered a wide range of taxa, though were mainly comprised of red, green and brown algae, as well as sponges (see supplementary data). As is common in screening work, DMSO was used to dissolve extracts at a maximum solvent concentration of 0.1% [24,25]. The low LDH cytotoxicity values for each treatment confirmed DMSO was tolerated by cells at this level, and indicated all extracts were non-toxic. The relatively low extract concentration may explain the limited effects seen on measures of cell activity–as neither hFOB proliferation nor MSC differentiation varied greatly from control values. Of those deviations seen, most were decreases in proliferation–whilst any increases were not large enough to imply future treatment potential. For differentiation, alkaline phosphatase (ALP) activity was investigated as it is an established early marker of cell differentiation—indicating osteoprogenitor cells are starting to form more mature osteoblasts [32]. However, no significant increases in ALP activity were seen with any extract treatment, indicating no stimulation of cell maturation.

As osteogenic activity was yet to be established a further 25 extracts were tested, though each was dissolved in ethanol rather than DMSO. As ethanol was tolerated up to a 0.5% level, this allowed a greater concentration of extracts to be tested with cells (approximately 150 μg/ml). However, despite this greater concentration extracts performed similarly to those dissolved in DMSO, mainly serving to decrease hFOB proliferation. One extract, E-05 (sea star, species unknown), did increase both hFOB proliferation and MSC differentiation–though not to a significant degree. This lack of activity is not surprising, as most similar screening exercises within this field only find a small number of active ‘hits’ in amongst many more inactive compounds. For example, one study carried out a high throughput chemical screen of 45,000 small molecules, finding only two series of small molecules with potent osteogenic activity [28].

The same extraction method, utilising dichloromethane and methanol, was used to produce material which was then dissolved in either ethanol or DMSO–producing final fractions for testing. As no substantial osteogenic potential was seen, the left-over material that did not dissolve in either dichloromethane or methanol–termed ‘powder extracts’ (due to their consistency)–was also tested. These extracts were added to treatment media as either a saline or water dissolved mixture, meaning solvent toxicity was not an issue and high extract concentrations could therefore be achieved. Powder extracts had little effect on hFOB proliferation, as with extracts dissolved in DMSO and ethanol. However, pronounced increases in MSC ALP activity with P-02 and P-04 treatments indicates these two extracts contain bioactives able to stimulate cell differentiation. P-02 extract was derived from the green seaweed species *Percursaria percusa* (C. Agardh) Rosenvinge, whilst P-04 was from the deep-sea sponge *Psolus squamatus* (O.F. Müller 1776). This positive result prompted further testing of seaweed, sponge and alga-derived powder material in both *in vitro* and *in vivo* assays, with one species in particular–the epiphytic red alga *Ceramium pallidum* (extract P-06)–showing strong bioactivity. Bioactivity for this species has been described in detail within the present study, to further highlight the osteogenicity of powder extracts and the importance of continued screening efforts.

There are limited reports mentioning *C*. *pallidum*, and none have investigated the osteogenic capacity of extracts from this species. MSCs were solely used for more detailed screening of promising extracts, as they are more representative of the cells within the human body [33], giving a better indication of treatment potential. Lack of proliferative effect separates *C*. *pallidum* extract from other algae-derived extracts, which do stimulate MSC proliferation, such as fucoidan [13] and the total algal extract from *Scytosiphon lomentaria* (Lyngbye) Link [34]. The increase of ALP activity in MSCs treated with 150 μg/ml *C*. *pallidum* indicates that the extract promotes cell differentiation at this optimum concentration. Lower levels had no effect, whilst the lack of activity seen with 1520 μg/ml indicates a complete suppression of ALP activity. This is unlikely to be related to toxicity, as proliferation was reduced but still evident at 1520 μg/ml. Instead it may indicate the presence of compound/molecule which inhibits differentiation at a high concentration. As previously mentioned, proliferation is often reduced during osteoblast differentiation and maturation [31], which may explain the lack of proliferative effect seen–an idea supported by similar work. For example, the aforementioned floridoside from the red algae *Laurencia undulata* Yamada had no significant effect on the degree of cell proliferation, but strongly increased cell differentiation and eventual mineralisation [35]. However, it should be noted that extracts which promote cell growth can still enhance differentiation, as with fucoidan [13].

Once mature, osteoblasts produce factors which control mineral production [36], leading to the formation of a mineralised extracellular collagen matrix and eventually new bone material [37]. Therefore, an extract which promotes differentiation would also be expected to enhance mineralisation level, as *C*. *pallidum* did in the present study. Here, extract inclusion caused a significant promotion of MSC mineralisation, particularly at 150 μg/ml, indicating early cell differentiation effects are maintained and lead to a greater degree of cell maturation by day 21. These results are again supported by similar reports of activity from osteogenic compounds and extracts, such as the aforementioned floridoside [35] and fucoidan. Fucoidan in particular is reported to cause promotions of ALP and mineralisation level that are tightly correlated [14,38]. Furthermore, whole extracts are also reported to stimulate osteoblast maturation, such as those derived from the halophyte plant *Salicornia herbacea* L. [39] and the brown algae *Sargassum horneri* (Turner) C.Agardh [40].

Based on *in vitro* data, *C*. *pallidum* appears to be a non-toxic extract, containing bioactive components able to stimulate osteoblast maturation and mineralisation. However, it was also important to assess if this osteogenic effect was maintained within a whole organism, to better indicate future treatment potential. Zebrafish was chosen as a model organism due to its many similarities with mammals regarding bone, including similar structures, regulators of bone formation and developmental processes [41]. Two systems were used to look at different aspects of skeletal formation: 1. an operculum-based system in larvae to measure bone growth, and 2. a fin-regeneration model in adult fish to measure bone regeneration. Firstly, the operculum system is particularly reliable and robust, allowing rapid detection of osteogenic, anti-osteoporotic and osteotoxic activity [20]. In the present study, one large mortality event did occur 72 hours post treatment in the 150 μg/ml group. Whilst concerning, this event is unlikely to indicate extract toxicity, considering it was limited to one treatment well and not seen when individuals were exposed to higher concentrations. Instead it is likely due to experimental mishandling and this treatment will be repeated in future studies to rule out any toxic effects of this particular concentration. Otherwise, *C*. *pallidum* had a very notable effect on operculum growth, causing large size increases over the 3-day growth period. This correlates well with *in vitro* data, though a higher concentration was required to fully promote operculum growth. However, this was understandable as treatments were applied systemically, meaning they had to be ingested or adsorbed through the skin [42]. Interestingly, even low concentrations still gave an effect equal to, or exceeding, the positive control calcitriol–a known enhancer of operculum [20] and skeletal growth [43]. The outperformance of calcitriols osteogenic effect by *C*. *pallidum* extracts demonstrates the strength of its anabolic effect, suggesting its osteogenic response may be maintained in mammals–such as rat and rabbit models of bone formation.

Adult individuals did not respond as obviously to *C*. *pallidum* treatment as larvae, though measurements of pixel intensity indicated brighter pixels and therefore a stronger staining in bony rays regenerated in fish exposed to the red algae extract [21]. This indicates that, relative to control, *C*. *pallidum* extract treatment was promoting the bone density of the regenerated rays, correlating with the previous *in vitro* and *in vivo* operculum effects seen. The slight increase in osteogenesis and decrease in regeneration supports this effect, though these were not significant in size. The reduced response in adults is likely due to differences in exposure method, as individuals were injected with treatments rather than being immersed in them. This injection was limited to a 3 μl volume per 100 mg fish weight, reducing the effective extract concentration that could be achieved. Furthermore, it meant individuals were treated only once during the 5-day regeneration period, rather than having constant exposure as with other uses of this model [21] and the larval operculum system.

In general, powder extracts were more successful than fractions dissolved in DMSO and ethanol, which may have been due in part to the use of a non-toxic solvent and the ability this afforded to use higher treatment concentrations. However, these fractions do contain different compounds and therefore it is also not unreasonable to suggest that it is something specific in their composition that is responsible for the increased bioactivity in these assays. As all powders were water soluble this could suggest the active component(s) was a polar molecule, such as a protein with exposed groups and a high dipole moment. If proteinaceous, bioactives in those powders treated with alkaline extraction would likely be degraded, forming small-chain peptides–or actives could instead be formed from more stable glycoproteins. Detailed chemical analysis would be required to confirm the bioactive component of promising extracts, though this was not undertaken as part of the current study. For screening efforts such analysis was unfeasible—given the number of extracts tested—and unnecessary, as none displayed significant osteogenic activity. For *C*. *pallidum*, some preliminary chemical analysis has been conducted–which indicated protein and a low level of polysaccharides to be present in the extract. Red algae are mainly comprised of protein (up to 47% of their dry matter), and often contain high mineral and polysaccharide levels [44]. Though red algal-derived minerals–like Aquamin–are known to have an osteogenic effect, *C*. *pallidum* is a non-mineralising species [45]. Furthermore, red algal cell wall sulphated polysaccharides also have no reports of osteogenic potential [46]. This leaves proteinaceous material most likely as an active component, or perhaps a small-chain peptide, glycoprotein or even a mixture of different bioactives. Future clinical use of this bioactive material is feasible, considering the worldwide abundance of the *Ceramium* genus [47], its fast growth rates and the relative ease of sourcing these shallow water eukaryotes.

## Conclusions

This work detailed screening efforts focused on discovering novel osteogenic potential in extracts derived from Irish marine organisms. Despite testing many extracts dissolved in DMSO and ethanol it was extraction of powder residue material which showed most promise–highlighting the need for continued screening efforts from different sources. Powder extract from *C*. *pallidum* in particular showed excellent *in vitro* and *in vivo* osteogenic potential, and may present a future therapeutic option for the treatment of osteoporosis and other musculoskeletal conditions. Further work will involve elucidating the exact bioactive component of this extract and its mechanism of effect.

## Supporting information

S1 Fig hFOB proliferation with DMSO dissolved extract set 1. Proliferation of hFOBs at day 4 **(a**) and 7 **(b**) calculated via PicoGreen assay. Cells were challenged with DMSO dissolved extracts D-01 to D-14. DMSO was included at 0.1%, giving extract concentrations ranging between 20 and 31.7 μg/ml. Cell proliferation, as a percentage of control, is presented as mean +/- SD, (n = 4). * indicates a statistically significant difference (*p*<0.05) compared to the relevant control.
(TIF)

S2 Fig hFOB proliferation with DMSO dissolved extract set 2. Proliferation of hFOBs at day 4 **(a**) and 7 **(b**) calculated via PicoGreen assay. Cells were challenged with DMSO dissolved extracts D-15 to D-28. DMSO was included at 0.1%, giving extract concentrations ranging between 20 and 31.7 μg/ml. Cell proliferation, as a percentage of control, is presented as mean +/- SD, (n = 4). * indicates a statistically significant difference (*p*<0.05) compared to the relevant control.
(TIF)

S3 Fig hFOB proliferation with DMSO dissolved extract set 3. Proliferation of hFOBs at day 4 **(a**) and 7 **(b**) calculated via PicoGreen assay. Cells were challenged with DMSO dissolved extracts D-29 to D-43. DMSO was included at 0.1%, giving extract concentrations ranging between 20 and 31.7 μg/ml. Cell proliferation, as a percentage of control, is presented as mean +/- standard deviation SD, (n = 4). * indicates a statistically significant difference (*p*<0.05) compared to the relevant control.
(TIF)

S4 Fig hFOB cytotoxicity results for DMSO dissolved extracts. Cytotoxicity results (LDH assay) for hFOBs at day 1. Cells were challenged with DMSO dissolved extracts D-01 to D-14 **(a)**, D-15 to D-28 **(b)** and D-29 to D-43 **(c)**. DMSO was included at 0.1%, giving extract concentrations ranging between 20 and 31.7 μg/ml. Cell death is presented as mean +/- SD (n = 4). * indicates a statistically significant difference (*p*<0.05) compared to the relevant control.
(TIF)

S5 Fig hFOB proliferation results for ethanol dissolved extract set 1. Proliferation of hFOBs at day 4 **(a**) and 7 **(b**) calculated via crystal violet assay. Cells were challenged with ethanol dissolved extracts E-01 to E-13 or control (EtOH). Ethanol was included at a 0.5% concentration, giving extract concentrations of 150 μg/ml. Cell proliferation, as a percentage of control, is presented as mean +/- SD, (n = 4). * indicates a statistically significant difference (*p*<0.05) compared to the relevant control.
(TIF)

S6 Fig hFOB proliferation results for ethanol dissolved extract set 2. Proliferation of hFOBs at day 4 **(a**) and 7 **(b**) calculated via crystal violet assay. Cells were challenged with ethanol dissolved extracts E-14 to E-25 or control (EtOH). Ethanol was included at a 0.5% concentration, giving extract concentrations of 150 μg/ml (except for E-19: 20.8 μg/ml and E-22: 86 μg/ml). Cell proliferation, as a percentage of control, is presented as mean +/- SD, (n = 4). * indicates a statistically significant difference (*p*<0.05) compared to the relevant control.
(TIF)

S7 Fig hFOB cytotoxicity results for ethanol dissolved extracts. Cytotoxicity results (LDH assay) for hFOBs at day 1. Cells were challenged with EtOH dissolved fractions E-01—E13 **(a)** and E-14—E-25 **(b)**. Ethanol was included at a 0.5% concentration, giving extract concentrations of 150 μg/ml (except for E-19: 20.8 μg/ml and E-22: 86 μg/ml). Cell death is presented as mean +/- SD, (n = 4). * indicates a statistically significant difference (*p*<0.05) compared to the relevant control.
(TIF)

S1 Dataset Dataset providing all raw values used to create figures in this manuscript. Datasets are labelled according to the extracts tested, the type of data (i.e. *in vitro* or *in vivo*), time-point used and which figure the data relates to.
(XLSX)
