# Correction: Screening for osteogenic activity in extracts from Irish marine organisms: The potential of *Ceramium pallidum*

**DOI:** 10.1371/journal.pone.0211395

**Published:** 2019-01-23

**Authors:** Matthew A. Carson, John Nelson, M. Leonor Cancela, Vincent Laizé, Paulo J. Gavaia, Margaret Rae, Svenja Heesch, Eugene Verzin, Brendan F. Gilmore, Susan A. Clarke

There is an error in affiliation 6 for author Margaret Rae. Affiliation 6 should be: Marine Institute, Rinville, Oranmore, Co. Galway, Ireland.
