# Supplementary figures and images for: Screening for osteogenic activity in extracts from Irish marine organisms: The potential of Ceramium pallidum
Source: PLoS One. 2018 Nov 28;13(11):e0207303. doi: 10.1371/journal.pone.0207303 (PMC6261572; doi:10.1371/journal.pone.0207303)

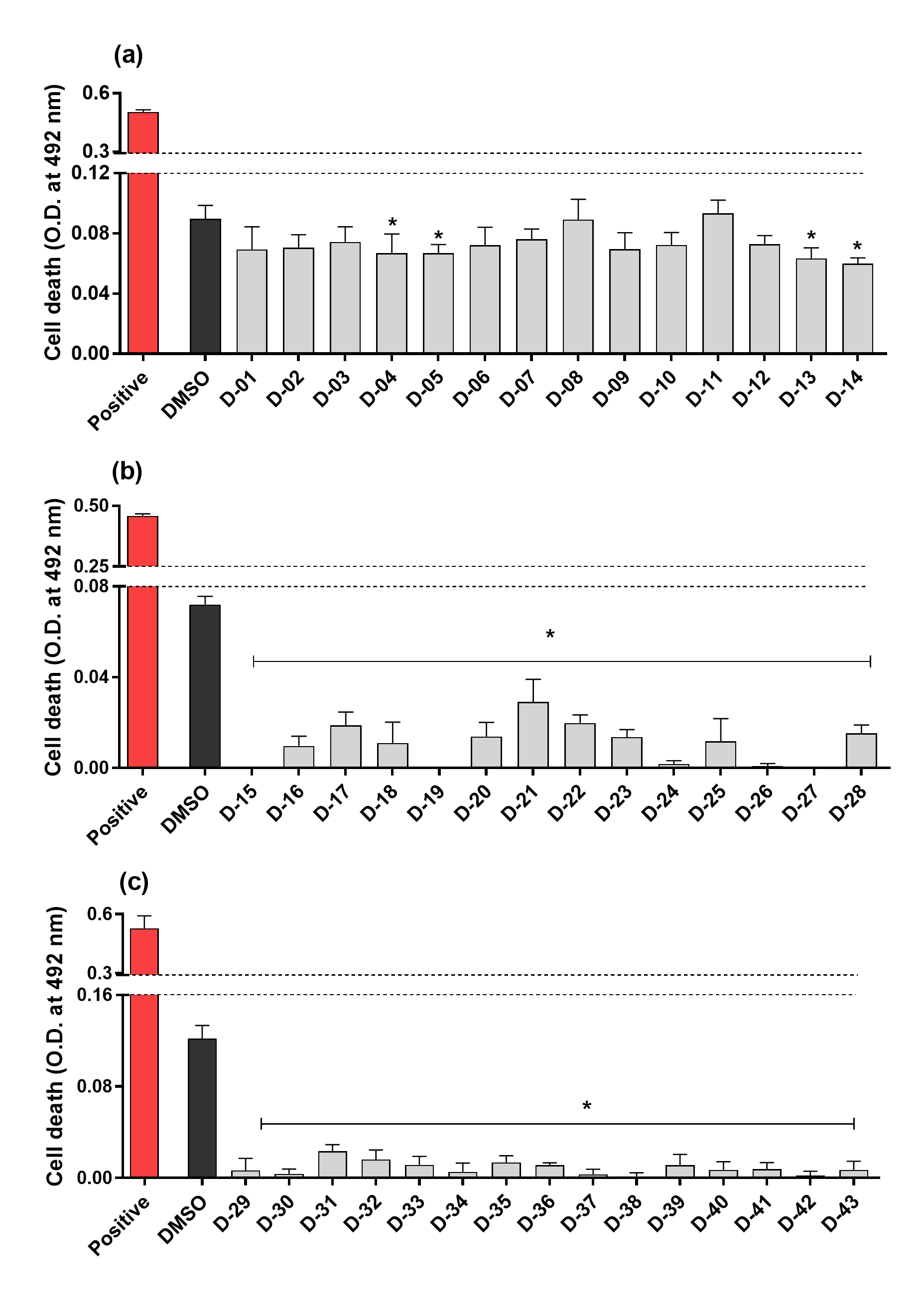

Supplement: S4 Fig — Cytotoxicity results (LDH assay) for hFOBs at day 1. Cells were challenged with DMSO dissolved extracts D-01 to D-14 (a), D-15 to D-28 (b) and D-29 to D-43 (c). DMSO was included at 0.1%, giving extract concentrations ranging between 20 and 31.7 μg/ml. Cell death is presented as mean +/- SD (n = 4). * indicates a statistically significant difference (p<0.05) compared to the relevant control. (TIF) [file pone.0207303.s004.tif]
